# A New Medical School

**Published:** 1850-07

**Authors:** 


### A NEW MEDICAL SCHOOL.

It may not be unknown to our readers that a prospectus for a new medical school in this city has been published in the newspapers, and in some of the medical journals. The teachers consist of a portion of the Faculty of the Lexington medical school, and of several medical gentlemen of this city. In view of the fact that the Kentucky Legislature had refused to grant a charter for another medical school in Louisville, the announcement that a new school, "*under the auspices of the Masonic University,*" would be opened in Louisville, caused not a little surprise, and more than ordinary speculation. Much curisosity was manifested to see the charter of the Masonic University, an institution located in Lagrange, Oldham county. It was believed that a law passed in 1834, passed too at the instance of the Lexington school, which prohibits any college from branching away from its own location, would stand in the way of this new enterprize. We were, therefore, curious on these points, but were unable to obtain any satisfaction on the subject until a controversy sprung up on a proposition to admit the new school teachers to the privileges of the hospital. This controversy caused a publication of the charter of the Lagrange College, and also the statutory provision already referred to. This publication was made in the *Louisville Daily Journal*, and in its columns we saw for the first time the charter of the College, and the prohibitory law. In order to give our readers a correct understanding of the subject, we republish the charter and the law:

### AN ACT TO AMEND AN ACT ENTITLED AN ACT TO INCORPORATE FUNK SEMINARY.

SECTION 1. *Be it enacted by the General Assembly of the Commonwealth of Kentucky*, That the charter of Funk Seminary, situated at Lagrange, in Oldham county, be so amended as to vest in the trustees of said institution all the powers which by law pertain to a university; and that said institution be entitled, in addition to its present name, to that of the Masonic University of Kentucky, and said trustees, by that name, be treated a body politic and corporate, with such successions as are provided for in the existing charter and with power to hold necessary real and personal estate, contract and be contracted with, sue and be sued, implead and be impleaded, both at law and equity in the courts of this Commonwealth.

SEC. 2. That said trustees and their successors shall and may at all times hereafter be capable in law to have, hold, receive, take, and retain, to them and their successors forever, for the use and benefit of said University, any property, real, personal, or mixed, by purchase, gift, grant, devise, bequest, or otherwise, in trust or in fee simple from any person or persons, bodies corporate and politic, provided always, that the amount shall be so limited as that the annual income thereof to the said University shall not exceed $50,000.

SEC. 3. That the president and trustees of said University shall have power to confer degrees in any and all the faculties, arts, sciences, and liberal professions, and also the honorary degrees usually conferred in any of the colleges and universities of the United States.

SEC. 4. That the said University, recognizing the being and government of Almighty God, shall be founded and continued on the plan of the great social and political institutions of the United States, having respect to the liberal and enlightening principles on which they are founded, and that no laws, rules, or regulations of a sectarian or party character, either in religion or politics, shall ever be adopted or imposed by which any student shall be subjected or made liable to any disabilities or disadvantages whatever on account of his political or religious opinions.

SEC. 5. That the president, with the consent and approbation of said trustees, shall have power to confer the title of Magister Docendi upon such students as, upon examination in the presence of the trustees or a committee by them appointed, shall be found qualified to act as teachers and shall be found worthy of the honor. That all diplomas shall be signed by the president and trustees or a committee of such trustees under the corporate seal of said University.

SEC. 6. That instruction, both theoretical and practical, shall be furnished at the said University in the art of school keeping, and a register of the names of persons desiring to become teachers in the State of Kentucky shall be kept in the library of the said University, and all who may wish to employ teachers shall have access to the said register, together with all the particulars pertaining to the qualifications of the candidate for teaching.

SEC. 7. That the president of the said University shall nominate, and by and with the advice and consent of the board of trustees, shall appoint as many professors and teachers as the trustees may deem necessary to advance the cause of education and the welfare of the University.

THOMAS W. RILEY,
Speaker of the House of Representatives.
JOHN L. HELM,
Speaker of the Senate.

Approved, March 4, 1850.

J. J. CRITTENDEN.

I, John W. Finnell, Secretary of State for the Commonwealth of Kentucky, do hereby certify that the foregoing manuscript contains a true copy of an enrolled bill on file in my office, entitled, "An act to amend an act entitled an act to incorporate Funk Seminary."

In testimony whereof, I have hereunto subscribed my name and affixed the seal of my office, at Frankfort, this 15th day of June, 1850, and of the Commonwealth the 59th.

JOHN W. FINNELL,
Secretary of State.

The following is the law alluded to: approved the 24th of February, 1834. The section reads as follows:

It shall not be lawful for said Theological Seminary to establish any other branches of learning than that herein provided for, nor shall it be lawful for it *or for any other college, seminary, or university in this State to constitute professorships or establish any branch of learning out of the county in which such seminary, college, or university may be located, and all such so established are to be taken and considered as against law and void.*

It is denied that this charter gives any power to confer degrees in medicine, and eminent lawyers concur in this view of the case. But on the other hand, the friends of the new school affirm that they have the written opinion of legal gentlemen, which asserts the existence of the power in the charter. It seems to us that the opinion, and the names of its holders should be published—there could be no better time for this publication, than when the contrary view is loudly and broadly proclaimed.

In reference to the statutory prohibition we have quoted above, the advocates of the new school deny that the Masonic University "has done, or attempted to do, the questionable act." Again, it has been authoritatively declared, that if inquiry were made of the trustees of the Masonic University, it would be found "that the Masonic University has not established a branch or department in the city of Louisville either nominally or actually, and moreover, that at the present time the trustees contemplate no such enormity." We quote this from an authoritative statement on the subject, by an advocate of the new school, and in the absence of such branching power, the repudiation of all intention of the kind, at present, and the refusal of the Legislature to grant a charter, we are at a loss to know what the legal powers of this Association are.

